# A Case of Multiple Dural Arteriovenous Fistula-Like Findings Following Aneurysmal Subarachnoid Hemorrhage

**DOI:** 10.7759/cureus.100296

**Published:** 2025-12-28

**Authors:** Sujong Pak, Taisuke Akimoto, Wataru Shimohigoshi, Arisa Umesaki, Osamu Masuo, Tetsuya Yamamoto

**Affiliations:** 1 Neuroendovascular Surgery, Yokohama Municipal Citizen's Hospital, Yokohama, JPN; 2 Neurosurgery, Yokohama City University School of Medicine, Yokohama, JPN

**Keywords:** davf-like finding, direct physiological arteriovenous pathways, endovascular treatment, intracranial hypertension, sah

## Abstract

Dural arteriovenous fistula (dAVF) is thought to arise from triggers like venous sinus thrombosis or trauma, though many aspects remain unclear. We report the case of a 48-year-old woman who developed multiple asymptomatic dAVF-like findings two weeks after coil embolization for subarachnoid hemorrhage (SAH). Initial angiography showed no dAVFs. Postoperative angiography showed cavernous sinus and middle meningeal artery dAVF-like findings without cortical venous reflux. Conservative management was chosen, and follow-up angiography at three months showed spontaneous resolution. The incidental emergence of multiple dAVF-like findings suggests that increased intracranial pressure due to SAH may have opened “direct physiological arteriovenous pathways”, contributing to the development of dAVF. To the best of our knowledge, no previous reports have described a case presenting dAVF-like findings within a short period (two weeks) following an uneventful endovascular treatment. This case provides important insights into the existence and activation of “direct physiological arteriovenous pathways” and may contribute to a better understanding of the pathophysiology and mechanisms underlying dAVF development in human.

## Introduction

A dural arteriovenous fistula (dAVF) is an abnormal direct connection between dural arteries and dural venous sinuses or cortical veins, bypassing the normal capillary bed. These lesions are typically acquired and can lead to altered venous drainage, increased venous pressure, and, in some cases, neurological symptoms depending on the pattern of venous outflow [1]. The pathogenesis of dAVF is thought to be triggered by factors such as venous sinus thrombosis, trauma, and craniotomy, but many aspects remain unclear [2].

Although the exact etiology is unknown, venous hypertension is considered a major factor, and animal studies have shown that elevated venous pressure can open “direct physiological arteriovenous pathways” present in the dura. Furthermore, experimental studies have demonstrated that increased angiogenic factors themselves contribute to dAVF formation [3]. In animal experiments, angiogenic factors were significantly elevated one week after procedures that increase venous pressure, which partially supports our observation of dAVF-like findings in this patient two weeks postoperatively [3]. Additionally, intracranial hypertension may directly induce the expression of angiogenic factors, potentially contributing to dAVF formation, and can also cause venous occlusion or impaired venous circulation, leading to elevated intracranial venous pressure and subsequent dAVF formation [4,5].

We report a rare case of a patient who developed multiple asymptomatic dAVF-like findings two weeks after coil embolization for a ruptured aneurysm causing subarachnoid hemorrhage (SAH). To the best of our knowledge, based on a literature search of PubMed database, no previous reports have described a case presenting dAVF-like findings within a short period (two weeks) following an uneventful endovascular treatment. Therefore, the clinical role of these mechanisms in humans remains uncertain. This case provides valuable insights suggesting that intracranial hypertension may have contributed to the development of dAVFs.

## Case presentation

A 48-year-old woman with no significant past medical or medication history was found collapsed at home and was transported to our hospital by ambulance. On arrival, her neurological status was Glasgow Coma Scale E2V3M5 [6], indicating marked disturbance of consciousness. No gross motor weakness was observed; however, aphasia was present.

**Figure 1:**
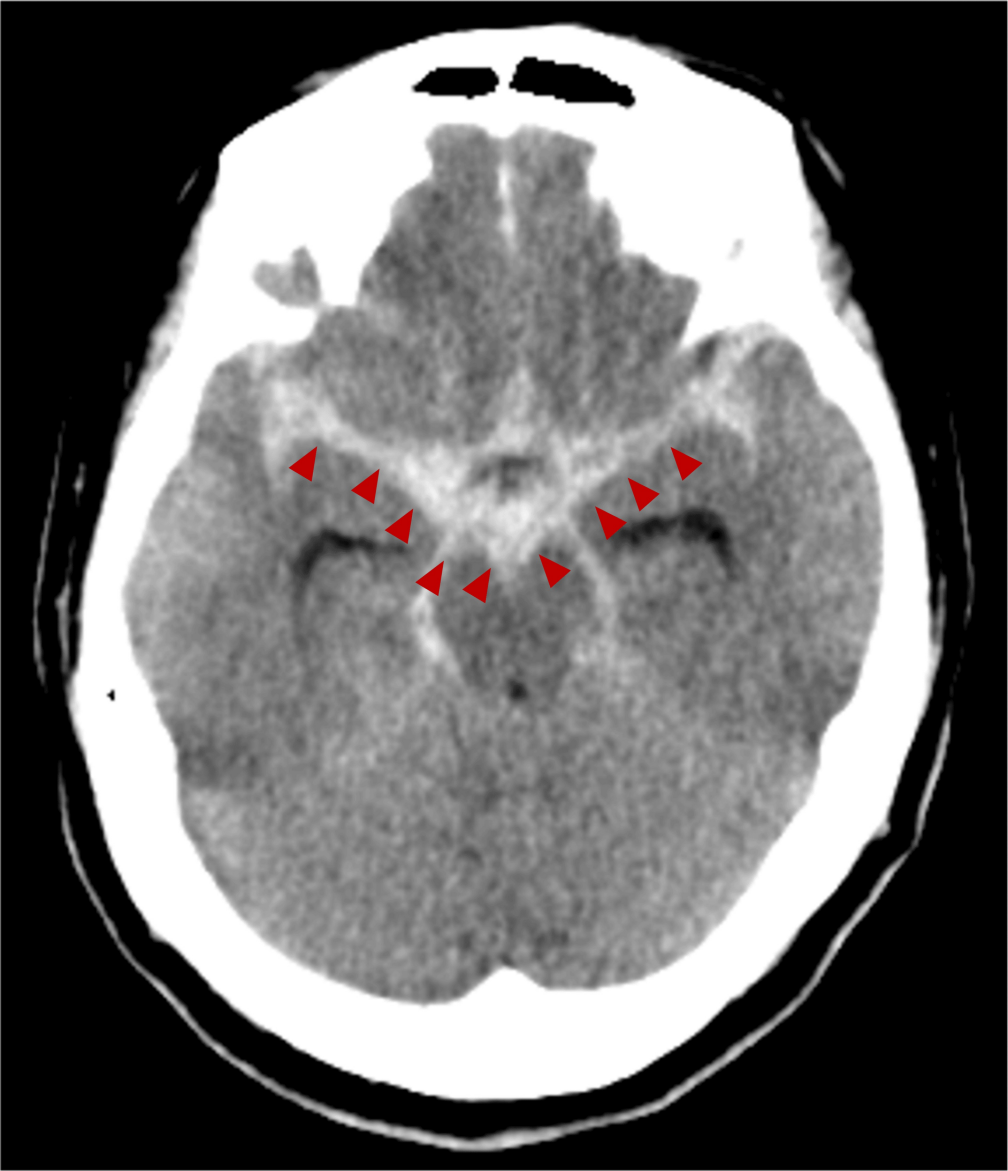
Initial CT Head at admission A thick hematoma was observed in the basal cistern (red arrow head), consistent with a diagnosis of sabarachnoid hemorrhage.

**Figure 2:**
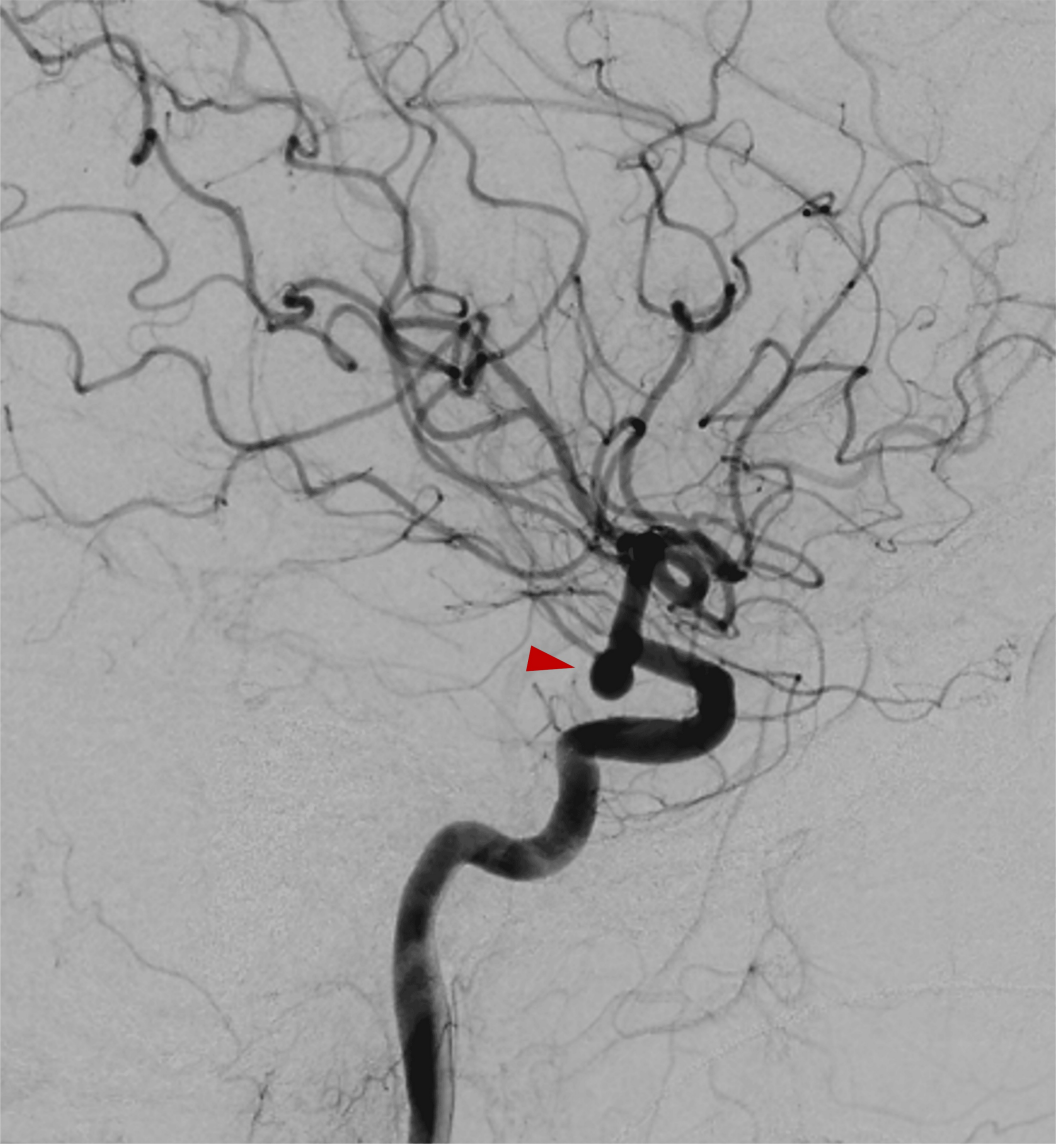
Preoperative angiography Right internal carotid–posterior communicating artery aneurysm (red arrow head) was considered the source of rupture.

**Figure 3:**
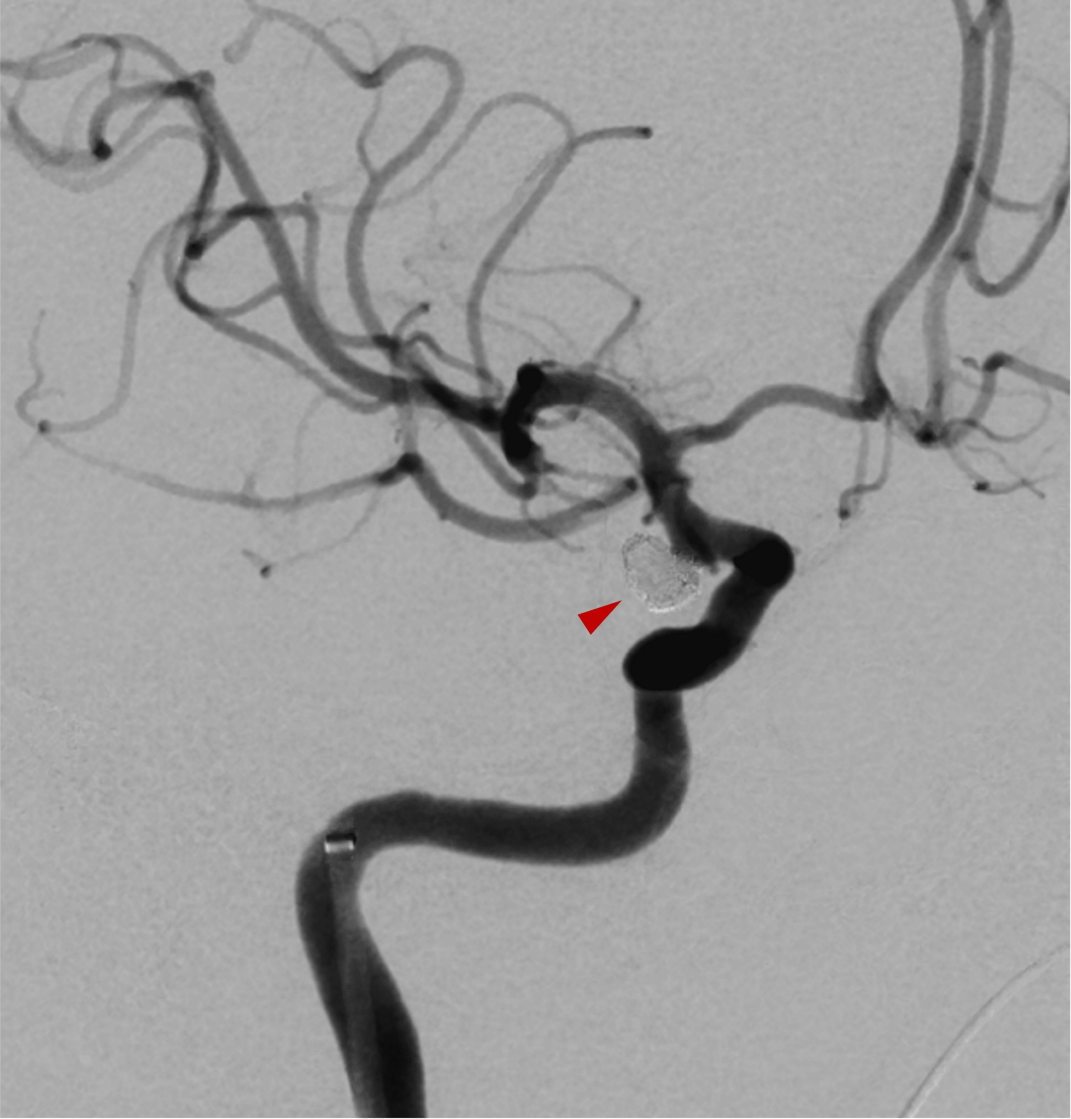
Angiography immediately after coil embolization Coil embolization for the internal carotid–posterior communicating artery aneurysm was performed using the single-catheter technique (red arrow head).

Computed tomography (CT) of the head revealed a thick hematoma in the basal cisterns (Figure 1). The World Federation of Neurosurgical Societies grade was 4, and the Fisher grade was 3. The cerebral angiography demonstrated an aneurysm at the right internal carotid-posterior communicating artery (IC-PC) junction, identified as the bleeding source (Figure 2). Coil embolization was performed (Figure 3). No abnormal vasculature, including dAVF, was observed on angiography.

**Figure 4:**
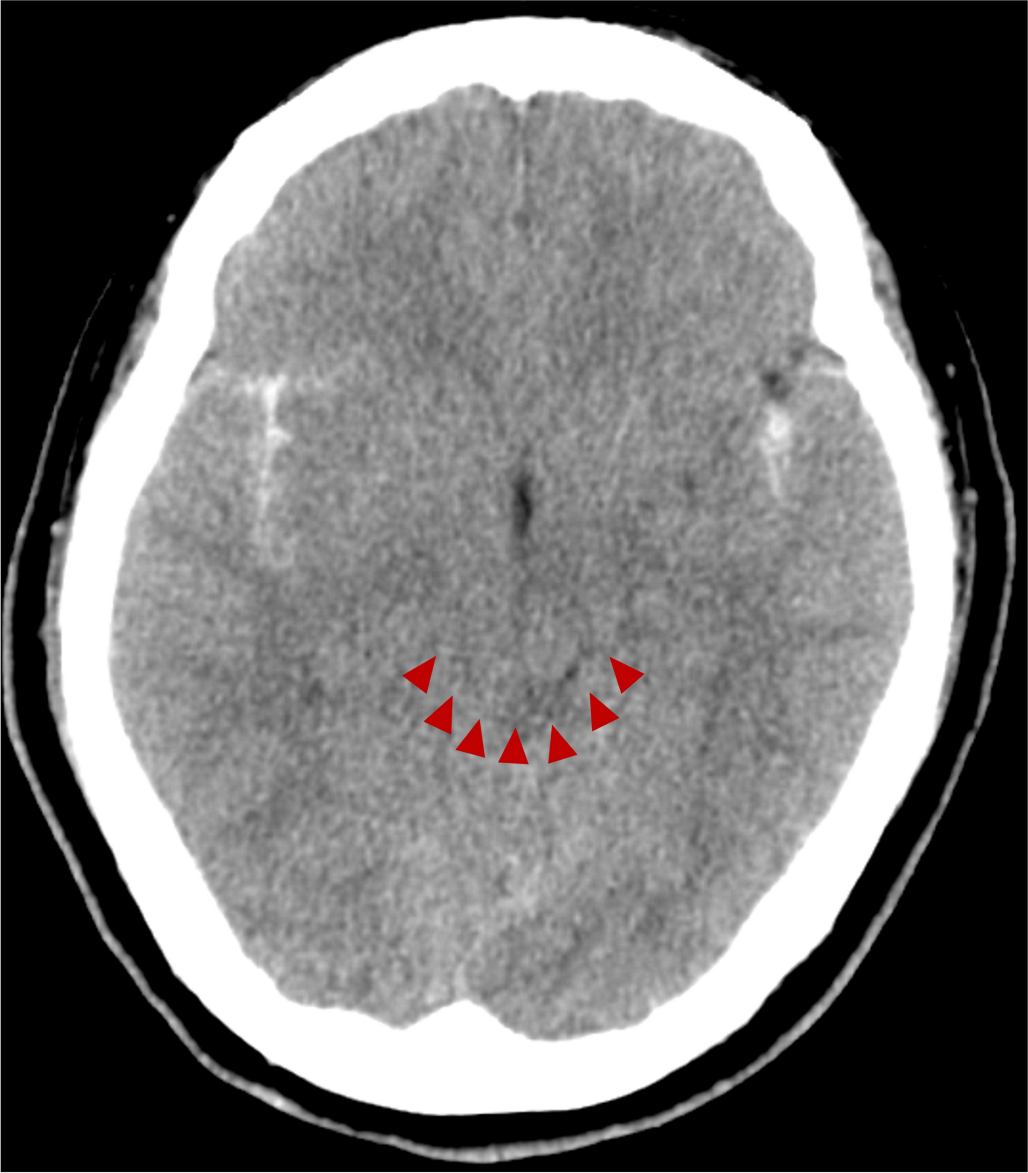
CT Head on postoperative day 1 Head CT showed narrowing of the ambient cistern and quadrigeminal cistern (red arrow head), suggesting increased intracranial pressure, and a lumbar drainage was placed.

**Figure 5:**
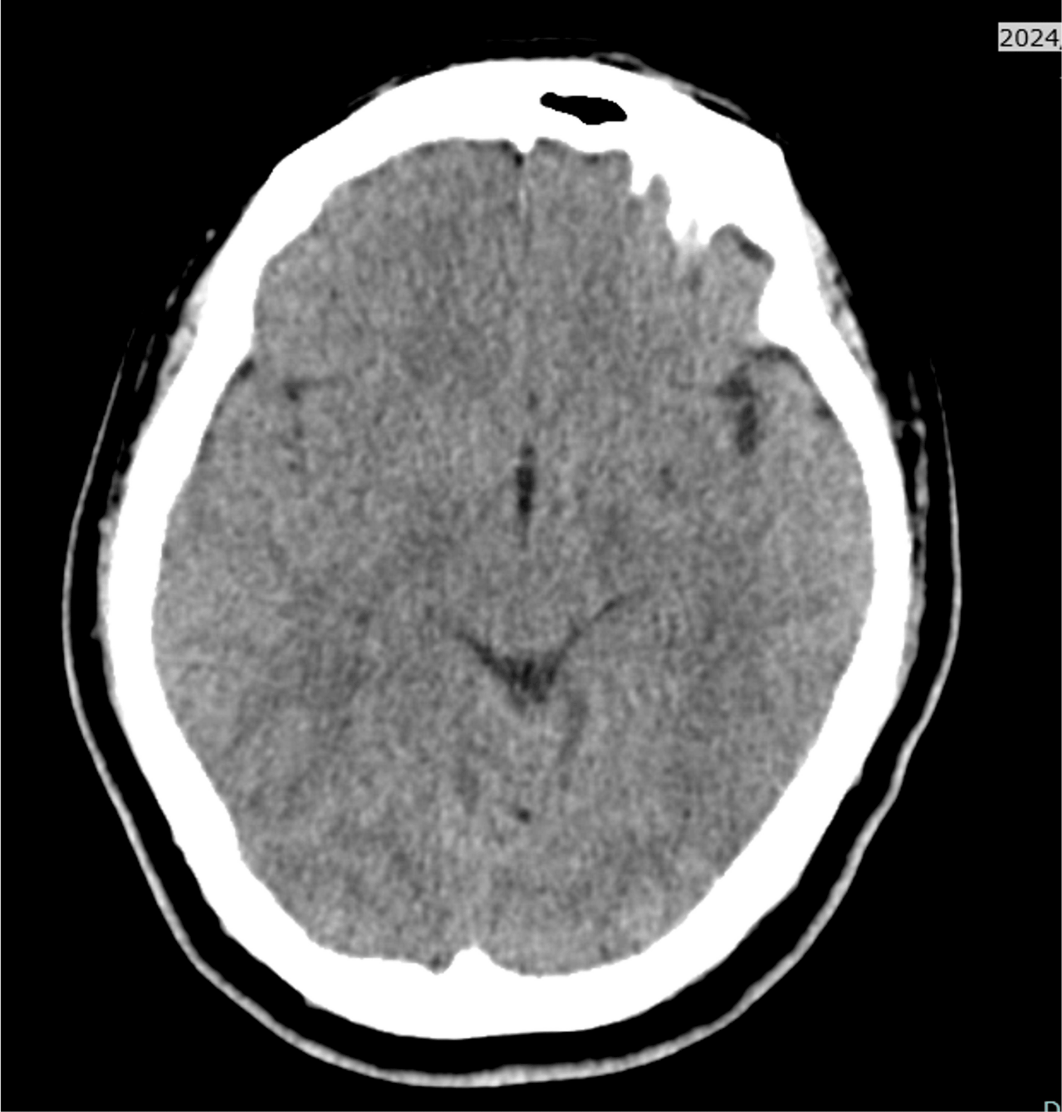
CT Head on postoperative day 4 Improved visualization of the basal cisterns was observed, suggesting resolution of intracranial hypertension.

On postoperative day 1, Head CT revealed ventricular narrowing and compression of the ambient cistern and quadrigeminal cistern, suggesting increased intracranial pressure (ICP) (Figure 4). A lumbar drain (LD) was placed, and the lumbar opening pressure was 11 cmH₂O. Although the opening pressure measured during lumbar puncture was not particularly high, the possibility that CSF flow was obstructed by the hematoma resulting from SAH was also considered. As the visualization of the basal cisterns improved, LD was removed on postoperative day 4 (Figure 5). 

**Figure 6:**
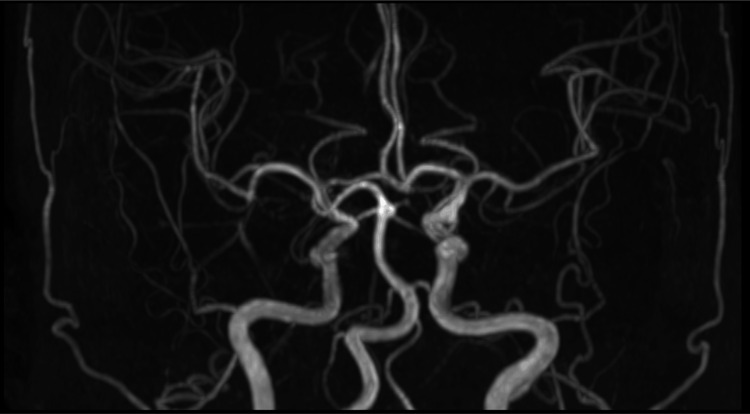
Magnetic resonance angiography at two weeks after surgery No evidence of cerebral vasospasm or abnormal vessels.

The patient’s level of consciousness gradually improved, and by one week after the procedure, the GCS score was E4V5M6. No obvious central neurological deficits were observed. Magnetic resonance imaging (MRI) performed one and two weeks postoperatively showed no clear evidence of cerebral vasospasm or abnormal vessels (Figure 6) and no hydrocephalus.

**Figure 7:**
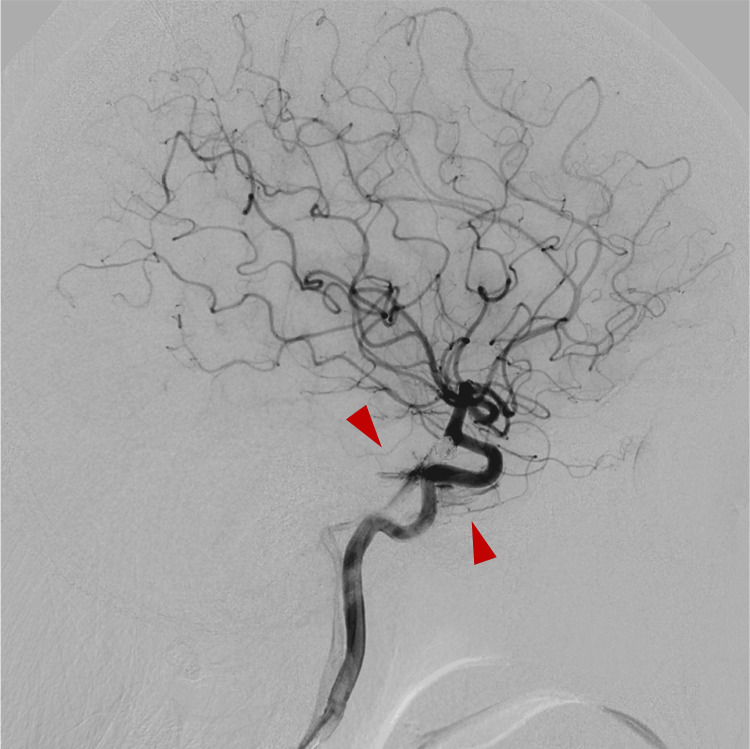
Cerebral angiography two weeks after embolization Right internal carotid artery angiogram demonstrated a cavernous sinus dural arteriovenous fistula-like findings, supplied by the dorsal meningeal artery and tentorial artery via the meningohypophyseal trunk, with drainage into the inferior petrosal vein.

At our institution, cerebral angiography is routinely performed two weeks after surgery, once the spasm period has passed, in all cases to evaluate for potential complications following coil embolization for a ruptured aneurysm. Cerebral angiography at two weeks after the operation showed multiple de novo dAVF-like findings. Right ICA angiography demonstrated a cavernous sinus dAVF (CSdAVF)-like feature fed by the meningohypophyseal trunk draining into the inferior petrosal vein (Figure 7).

**Figure 8:**
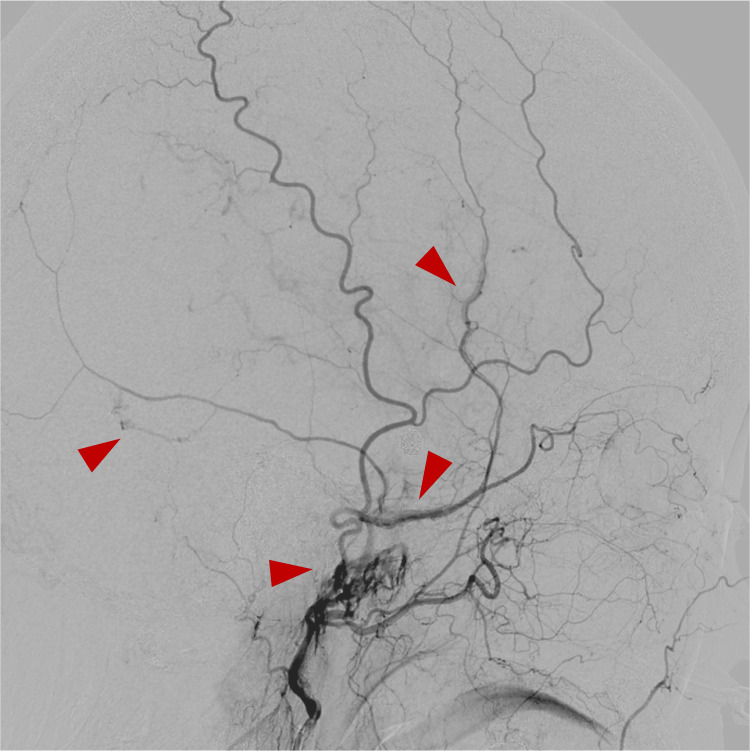
Cerebral angiography two weeks after embolization Right external carotid artery angiogram revealed multiple middle meningeal arteriovenous fistula-like findings (red arrow head), supplied by the main trunk of the MMA and draining into adjacent veins.

Right external carotid artery angiography showed multiple middle meningeal artery dAVF (MMAVF)-like findings draining into the middle meningeal vein (MMV) and pterygoid plexus (Figure 8). 

**Figure 9:**
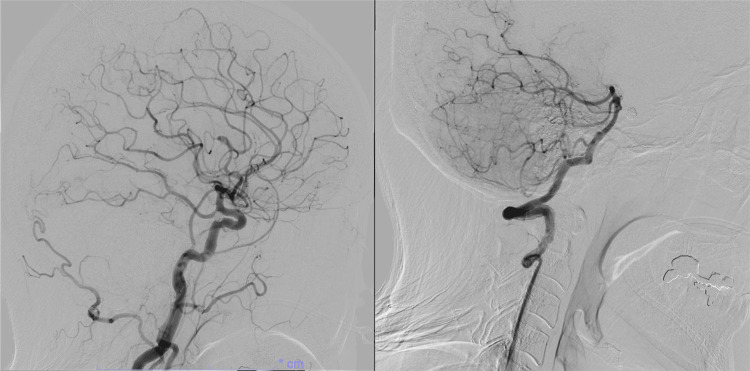
Cerebral angiography two weeks after embolization No abnormal vessels were observed on left internal carotid and vertebral artery angiography.

Cortical venous reflux was not observed. No apparent shunt vessels were observed on left common carotid artery angiography or left vertebral artery angiography (Figure 9). 

**Figure 10:**
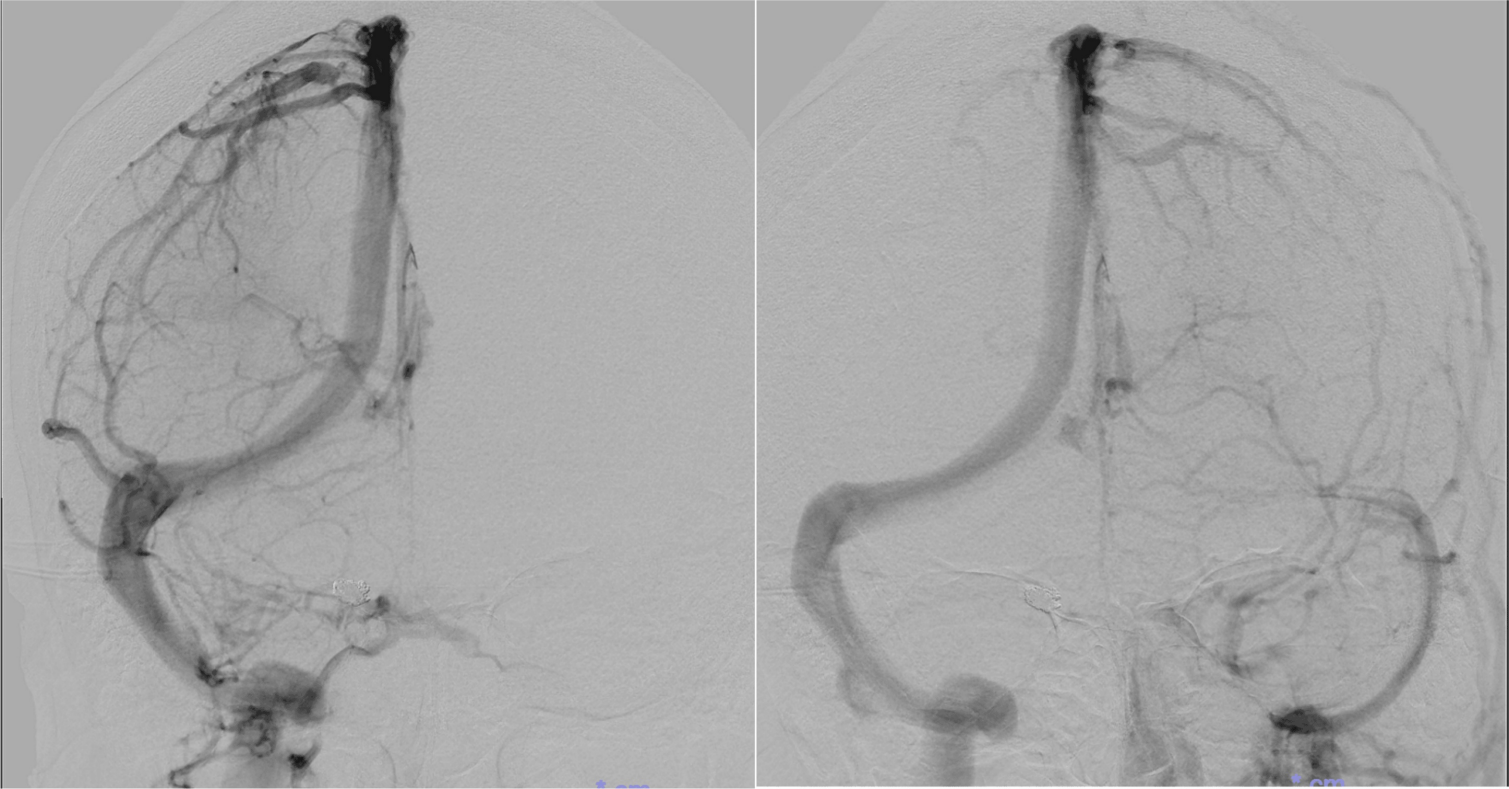
Venous phase images from cerebral angiography at the time of resolution of the dural arteriovenous fistula-like findings The transverse sinus was dominant on the right side.

Venous outflow analysis confirmed no venous occlusion. Regarding the venous drainage pattern, the anterior circulation drained predominantly into the right transverse sinus (Figure 10). 

The patient remained asymptomatic, and because the fistulas were Borden type I, conservative management was chosen. She was transferred with modified Rankin Scale score of 2, three weeks after surgery. Follow-up MRI and angiography three months later confirmed complete resolution of the shunts.

## Discussion

This case involved a patient who underwent coil embolization and subsequently developed dAVF-like findings following an episode of intracranial hypertension, which later spontaneously resolved. This rare case may provide insights into the potential involvement of intracranial hypertension in the pathogenesis of dAVF, though the observations should be interpreted as hypothesis-generating rather than definitive.

DAVFs are primarily considered acquired lesions caused by obstructive changes on the venous side, such as venous sinus thrombosis, trauma, or surgery [1,2]. Although the exact etiology remains unclear, venous hypertension is considered a principal factor, and experimental studies suggest that it can open latent "direct physiological arteriovenous pathways" within the dura [3]. These pathways are naturally existing microvascular connections between arteries and veins within the dura mater. Under normal conditions, they are closed or minimally perfused, allowing normal cerebral circulation without shunting [7]. Certain pathological stimuli, such as venous hypertension, may transiently open these pathways, resulting in abnormal arteriovenous shunts. In a rabbit model, elevated ICP was shown to play a key role in dAVF formation [3]. Increased ICP, reduced cerebral perfusion pressure, and resultant cerebral ischemia due to cerebral venous thrombosis or venous sinus occlusion led to the opening of these pathways within the dura, resulting in shunt formation [8]. This stage has been described as a "pre-dAVF stage," which remains a hypothetical concept; subsequent hypoxia induces angiogenesis and vascular remodeling, ultimately leading to the development of a dAVF. Studies in rat models indicate that these pathways are most frequently found in the dura adjacent to venous sinuses [3].

In the present case, multiple asymptomatic dAVF-like findings were observed two weeks after coil embolization for aneurysmal SAH. In animal experiments, angiogenic factors were significantly elevated one week after procedures that increased venous pressure, partially supporting our observation of dAVF-like findings two weeks postoperatively [3,4].

The patient exhibited a transient arteriovenous shunt without a definite venous pouch or cortical venous reflux. The shunt involved perfusion from the MMA to adjacent venous structures. Because these findings differed from conventional dAVFs classifiable by Borden or Cognard systems, we interpreted this as a reversible “pre-dAVF stage” rather than a true dAVF, and defined it as “dAVF-like findings” [1].

The patient experienced severe SAH, and CT demonstrated classical signs of elevated ICP, including sulcal effacement, blurring of the gray-white matter junction, and obliteration of the basal cisterns [9,10]. Subsequent angiography suggested that elevated ICP may have opened latent arteriovenous pathways, resulting in shunts located adjacent to venous sinuses such as the cavernous sinus and middle meningeal vein, consistent with prior experimental data. Additionally, intracranial hypertension itself has been reported to increase HIF-1α expression, which can be suppressed by decompression, supporting early ICP management [4]. In this case, early ICP relief via LD may have contributed to preventing progression to the vascular remodeling stage, allowing physiological shunts to close spontaneously; however, this remains speculative.

In animal experiments, angiogenic factors were significantly elevated one week after procedures that increased venous pressure. Although this remains speculative, a similar delayed increase in angiogenic factors may also occur in humans following intracranial pressure elevation. In those experiments, angiography was performed only at 90 days, confirming dAVF formation; thus, the transition from dAVF-like findings to a true dAVF may require approximately three months. These observations suggest that appropriate intracranial pressure management may play a role in preventing progression to a true dAVF [3].

The relationship between intracranial hypertension and shunt formation has been experimentally demonstrated, but its clinical significance in humans remains uncertain. Shunt formation may worsen venous congestion and ICP or, alternatively, serve as a compensatory mechanism to reduce venous pressure [11]. 

In this case, shunts developed only on the right side, consistent with right-dominant venous drainage. These pathways likely opened preferentially where venous outflow could be accommodated, potentially mitigating ICP elevation. While dAVF-like shunts may progress to definitive lesions under continued stimuli, timely ICP management, such as LD placement, may help prevent this progression, though this is speculative.

The patient received clazosentan for the prevention of cerebral vasospasm. Based on the complications reported for clazosentan in the literature [12], there is currently no evidence demonstrating an association with dAVF formation. However, the possibility cannot be entirely excluded, given that clazosentan is a relatively new medication. Furthermore, because the SAH-related hematoma may have contributed to thrombus formation within the cavernous sinus, additional evaluation, such as contrast-enhanced MRI to assess for microthrombi, would have been warranted.

This case provides insights into the possible existence and activation of direct physiological arteriovenous pathways within the dura and may contribute to understanding the pathophysiology and mechanisms underlying dAVF formation. Nevertheless, these pathways have not yet been directly demonstrated in humans, and further accumulation of similar cases is needed to draw definitive conclusions.

## Conclusions

We encountered the first case showing the transient opening of a “direct physiological arteriovenous pathway,” which may have formed in response to increased ICP. The observed dAVF-like findings are interpreted as a possible precursor stage to dAVF formation (“pre-dAVF stage”), which remains a hypothetical concept rather than an established entity. Although timely control of intracranial pressure may have contributed to preventing progression to a definitive dAVF in this case, this interpretation remains speculative. To date, no reports have described similar imaging findings in humans, and further investigation is required to clarify the role of these pathways and the underlying mechanisms of dAVF formation. This single case should therefore be regarded as a hypothesis-generating observation.
